# Volume Second

**Published:** 1881-01

**Authors:** 


### EDITORIAL DEPARTMENT.

"If thou hast truth to utter, speak;
And leave the rest to God."

VOLUME SECOND.—To the kind friends and patrons who granted their aid and encouragement during the first volume, and the many new ones we hope to add to the pleasant list as we journey along with the present one ; we desire to extend our congratulations and best wishes for their happiness and prosperity during 1881.

Our old friends understand our programme, and we will only add for the information of the new ones, that it is our purpose to render the INDEPENDENT PRACTITIONER a welcome visitor to all who wish to see our noble calling protected as far as possible from the degrading influences which have been gnawing and sapping its very vitals for a long while past. We intend also that it shall not fall below any medical journal of its size, in the amount and value of its reading matter, and that it shall be specially suited to the wants of the busy practitioner. A glance at the list of contributors, and the amount and value of the contents of Volume 1st, will satisfy those who may see the Journal for the first time, that we perform what we promise our readers we will do. Hoping for a large accession to our list of subscribers, and patrons, and again wishing all our readers abundant health and success for the new year, we enter with renewed hope and energy upon our duties for the second volume of the Journal.

